# Role of Cigarette Smoke on Angiotensin-Converting Enzyme-2 Protein Membrane Expression in Bronchial Epithelial Cells Using an Air-Liquid Interface Model

**DOI:** 10.3389/fphar.2021.652102

**Published:** 2021-03-30

**Authors:** Massimo Caruso, Alfio Distefano, Rosalia Emma, Michelino Di Rosa, Giuseppe Carota, Sonja Rust, Riccardo Polosa, Pietro Zuccarello, Margherita Ferrante, Giuseppina Raciti, Giovanni Li Volti

**Affiliations:** ^1^ Department of Biomedical and Biotechnological Sciences, University of Catania, Catania, Italy; ^2^ Center of Excellence for the Acceleration of Harm Reduction (CoEAHR), University of Catania, Catania, Italy; ^3^ Department of Clinical and Experimental Medicine, University of Catania, Catania, Italy; ^4^ Department of Medical, Surgical Sciences and Advanced Technologies “G.F. Ingrassia”, University of Catania, Catania, Italy; ^5^ Department of Drug Sciences, University of Catania, Catania, Italy

**Keywords:** ACE-2, nicotine, smoke, cigarette, epithelial cells

## Abstract

Prevalence studies of current smoking, among hospitalized COVID-19 patients, demonstrated an unexpectedly low prevalence among patients with COVID-19. The aim of the present study was to evaluate the effect of smoke from cigarettes on ACE-2 in bronchial epithelial cells. Normal bronchial epithelial cells (H292) were exposed to smoke by an air-liquid-interface (ALI) system and ACE-2 membrane protein expression was evaluated after 24 h from exposure. Our transcriptomics data analysis showed a significant selective reduction of membrane ACE-2 expression (about 25%) following smoking exposure. Interestingly, we observed a positive direct correlation between ACE-2 reduction and nicotine delivery. Furthermore, by stratifying GSE52237 as a function of ACE-2 gene expression levels, we highlighted 1,012 genes related to ACE-2 in smokers and 855 in non-smokers. Furthermore, we showed that 161 genes involved in the endocytosis process were highlighted using the online pathway tool KEGG. Finally, 11 genes were in common between the ACE-2 pathway in smokers and the genes regulated during endocytosis, while 12 genes with non-smokers. Interestingly, six in non-smokers and four genes in smokers were closely involved during the viral internalization process. Our data may offer a pharmaceutical role of nicotine as potential treatment option in COVID-19.

## Introduction

There is emerging evidence that both nicotine use and smoking status may have an impact on COVID-19 infection rates and hospitalizations, as well as disease severity and mortality. To this regard, some studies reported greater risk of severe complications and higher mortality rate in current smokers with COPD vs non-smokers, finding that smoking was most likely associated with the negative outcomes (Alqahtani et al., 2020; Zhang C. et al., 2020), although in these studies it is difficult to dissect the effects of smoking from those of the pre-existing pathologies of the subjects included in the study. Despite the worse outcomes observed in smokers affected by COVID-19, the effect of smoking on the risk of contracting SARS-CoV-2 infection remains debated. A systematic Review of the prevalence of current smoking among hospitalized COVID-19 patients in China demonstrated an unexpectedly low prevalence among patients with COVID-19, which was approximately 1/4th the population smoking prevalence (Farsalinos et al., 2020). Consistently, in a study of the Italian Veneto region, one of the most SARS-CoV-2 affected area in Italy, (Rossato et al., 2020) further demonstrated that patients' smoking history showed that none of their hospitalized patient was a current smoker, with 112 patients (84.8%) who had never smoked and 20 (15.2%) who were former smokers. The same authors also confirmed that there was no difference in the disease severity between patients who never smoked and former smokers. With respect to the conceivable molecular mechanisms underlying the possible association between smoking and viral infection, several reports focused their attention on the possible modulatory effect of smoking on ACE-2 protein expression which has been proved to serve as possible receptor for virus entry into epithelial cells (Walls et al., 2020). In addition, analysis of ACE-2 in SARS-CoV infected cells suggests that ACE2 is not only a receptor but is also involved in post-infection regulation, including immune response, cytokine secretion, and viral genome replication. Coronavirus entry into host cells is mediated by the homotrimeric *trans*-membrane spike (S) glycoprotein. Each S glycoprotein consist of two subunits: S1 and S2. The subunit S1 is responsible for virus binding to the host cell receptor ACE-2, otherwise the subunit S2 is responsible for fusion to the host cell membrane, and therefore for its entry into the host cell. When S glycoprotein is cleaved at the boundary between the S1 and S2 subunits, it changes to the state of non-covalently bound prefusion conformation. The S1subunit comprises the receptor-binding domain(s) that contributes to the stabilization of the prefusion conformation of the membrane-anchored S2 subunit containing the fusion machinery. S2 is further cleaved by host proteases immediately over the fusion peptide activating the protein for membrane fusion. As a result, coronavirus entry into susceptible cells is a complex process requiring the concerted action of receptor-binding and proteolytic processing of the S protein to promote virus-cell fusion (Walls et al., 2020). The importance of the molecular interaction between the viral S protein and ACE-2 is further highlighted by the discovery that soluble ACE-2 might binds to soluble receptor impairing SARS-CoV-2 entry (Batlle et al., 2020) to reduce the spread of COVID-19. Moreover, it was hypothesized that exogenous recombinant human (rh)ACE-2 may act as the “bait” to neutralize the spike protein on the surface of the SARS-CoV-2, thus inhibiting entry in host cells (Guo et al., 2020). Nevertheless, several discrepancies have been observed to this regard since some reports showed that smoking was associated with a significant increase of ACE-2 protein expression (Batlle et al., 2020; Guo et al., 2020). However, it should be taken into due account that in most cases no information on patients’ comorbidities and smoking habits are reported and in particular regarding *in vitro* studies and biopsies, the studies evaluated only total protein expression rather than its membrane localization (Leung et al., 2020a). The aim of the present study was to evaluate the effect of smoke derived from cigarettes in bronchial epithelial cells on ACE-2 membrane protein expression by using an *in vitro* exposure setting resembling those conditions experienced by smokers *in vivo* and assessing the effects by using a high content screening approach. Taken altogether, although the generalized advice to quit smoking as a measure to reduce health risk remains valid, the findings of the present study, together with the well-established immunomodulatory effects of nicotine, may offer a pharmaceutical role of nicotine as a potential treatment option in COVID-19 patients.

## Materials and Methods

To characterize human bronchial epithelial cells (H292) in terms of ACE-2 expression following cigarette smoke exposure, H292 cells were exposed to smoke by an air liquid interface (ALI) system, assessing target expression following them for 24 h from the exposure.

### Cell Culture

NCI-H292 cells were cultured in RPMI-1640 cell culture medium (supplemented with 2 mM l-glutamine, 10% fetal bovine serum (FBS), 50U/ml penicillin, and 50 μg/ml streptomycin) in a humidified atmosphere (5% CO2) at 37°C until 80% confluency were reached. 48 h before the exposure cells were trypsinized and seeded in 12 mm transwell inserts at the density of 3*105 cells/ml for 24 h with the same medium at the apical (0.5 ml) and basal (1 ml) sides. 24 h before the exposure basal and apical culture media were replaced with 1 ml and 0.5 ml respectively of UltraCULTURE™ media supplemented with 2 mM l-glutamine, 50 U/ml penicillin, and 50 μg/ml streptomycin.

### Cell Exposure to Smoke at Air-Liquid Interface

Reference tobacco cigarettes 1R6F (Kentucky University) were conditioned for at least 48 h in accordance to ISO 3402:1999 (https://www.iso.org/standard/28324.html) at 60 ± 3% relative humidity and 22 ± 1°C and then smoked under Health Canada Intense (HCI) regime (55 ml puff-volume, 2 s puff-duration, 30 s puff-frequency, bell-shaped puff-profile, with hole vents blocked) by a LM1 smoking machine (Borgwaldt KC GmbH, Hamburg, Germany). Air-liquid-interface (ALI) exposure of cells was obtained by an exposure system consisting in a perspex chamber sealed by O-rings and containing a bottom part filled with D-MEM high glucose media (20 ml) and an upper part without media (Strzelak et al., 2018; Changeux et al., 2020; Zhang T. et al., 2020) and with two polyurethane tubes connected for loading and discharging of the smoke (Supplementary Figure S1). Briefly, the culture medium is removed from the upper part of the transwell inserts which are placed in the exposure chambers on a support that allows the cells to remain basally wet with medium and to be exposed to the smoke/air apically by the LM1 smoking machine. Cells were exposed to 2, 5, 10, 15, and 20 puffs of smoke and to 20 puffs of room filtered air, as a control, in a different experimental run. Moreover, we maintained a transwell with the culture media in the upper side (INC) and another without the upper media (ALI) in the incubator (5%CO2; 37°C) as additional controls.

### Nicotine Dosimetry

Nicotine dosimetry was performed in media contained in the exposure system. Calibration range was between 1-50 μg/ml (1, 2, 5, 10, 20, 50 μg/ml). 1 ml of each sample and each calibration standards were added with nicotine-(methyl-d3) solution - used as internal standard at 100 μg/ml. After, 1 ml of 0.1 M sulfuric acid and 5 ml of acetonitrile were added to each one and they were vortexed and centrifuged at 2500 RCF for 4 min. Afterward, 1 ml of each sample was passed through a filter (mm 25; um 0.45) and transferred to a vial with a 250 μl conical insert. Nicotine was determined by UPLC-ESI-TQD (Waters Acquity), operating in multiple reaction monitoring (MRM) and positive ion mode. MRM transitions monitored (m/z) with cone and collision voltages are reported in supplementary materials (Supplementary Table S1). An Acquity UPLC® HSS T3 1.8 μm—2.1 × 100 mm column was used. Isocratic elution (80% water and 20% acetonitrile, both added at 0.1% with formic acid) was performed. The mass spectrometry settings were as follows: capillary energy at 3.0 kV, source temperature at 150°C, column temperature at 40°C, desolvation temperature at 500°C, desolvation gas at 1000 L/h and cone gas at 100 l/h.

### High-Content Screening and Image Analysis

At the end of each exposure/treatment, H292 cells were trypsinized, counted and seeded in a 96-well (CellCarrier™-96; PerkinElmer #6005550) in triplicate at the density of 1*10^3^ cells/well and then placed in the incubator (5%CO_2_; 37°C) for 24 h. Then cells were labeled for nuclei (NucBlue™ Live cell Stain, Thermo-Fisher Scientific #R37605), membranes (CellMask™ Green Plasma Thermo-Fisher #C37608) and ACE-2 protein receptor (primary antibody: mouse anti-hACE-2; R&D Systems, #MAB933. Secondary antibody Alexa Fluor™ 546 goat anti-mouse IgG; thermo Fisher Scientific, #A11003). Membrane protein expression was assessed by High Content Screening (HCS) analysis using the PerkinElmer Operetta High-Content Imaging System. A dose-response curve and IC50 for ACE-2 expression were calculated, related to both nicotine in the exposed basal media and puff number. Air exposure and incubator controls were used for all the experiments. HCS analysis of ACE-2 protein expression was evaluated following membrane segmentation. Plates were read under confocal conditions using the 20x long WD objective. Enough fields were imaged to capture at least 500 cells/well. All images were analyzed using Harmony high-content imaging and analysis software (PerkinElmer). Final output values from the analysis were expressed as mean fluorescence intensity (MFI) percentage of control per well.

### IL6 and IL8 Concentration Enzyme-Linked Immunoassay

The concentration of IL6 and IL8 cytokines were measured in UltraCULTURE™ medium used for a 24 h cell recovery after the smoke exposure by the Human IL-6 Instant ELISA Kit and Human IL-8 Instant ELISA Kit (Invitrogen™). Each exposure condition was assayed in duplicate. The assays were carried out following the manufacturers’ instructions.

### Bioinformatic Analysis

#### Dataset Selection and Study Population

In order to test our hypotheses, we selected microarray dataset of small airway epithelium (SAE) of age matched “healthy” nonsmokers and “healthy” smokers without lung disease symptoms. The GSE52237 transcriptome dataset selected was downloaded from NCBI Gene Expression Omnibus (GEO) database (http://www.ncbi.nlm.nih.gov/geo/) (Clough and Barrett, 2016). Mesh terms “epithelial cells”, “smokers”, and "human", was used to identify potential datasets of interest. The clinical characteristics of all samples obtained from the GSE52237 dataset are already available (Walters et al., 2014).

The criteria for “healthy” was based on history, physical exam, complete blood count, coagulation studies, liver function tests, urine studies, chest X-ray, EKG and pulmonary function tests reported by the authors of original dataset analysis (Walters et al., 2014). Smoking status was verified by urine nicotine and cotinine levels (nonsmokers with urine nicotine <2 ng/ml and cotinine <5 ng/ml, smokers with urine nicotine ≥30 ng/ml and/or urine cotinine ≥50 ng/ml). Small airway epithelium (SAE) (10th to 12th order) was collected by fiberoptic bronchoscopy by brushing as reported by a previous paper published by the authors of the original dataset deposited (Harvey et al., 2007).

#### Dataset Processing

In order to process and identify Significantly Different Expressed Genes (SDEG) in dataset, we used the MultiExperiment Viewer (MeV) software. In cases where multiple genes probes insisted on the same GeneID, we used those with the highest variance. The significance threshold level was *p* < 0.05. Statistically significant genes will be selected for further analysis. We performed a statistical analysis with GEO2R, applying a Benjamini&Hochberg FDR (False discovery rate) to adjust *p* values for multiple comparisons.

Gene’s annotation was obtained by STRING software (https://string-db.org/). The STRING-combined score was based on data from neighborhood in the genome, gene fusions, co-occurrence across genomes, co-expression, experimental/biochemical data, and association in curated databases (Szklarczyk et al., 2017). The genes overlapping was represented with Venn diagram using a public online tool (http://bioinformatics.psb.ugent.be/webtools/Venn/) and the images readapted for our data with CorelDraw.

### Statistical Analysis

For statistical analysis, Prism 8.0.2 software (GraphPad Software, USA) was used. Shapiro-Wilk test was used to assess the symmetry of data distribution. Significant differences between groups were assessed using the one-way ANOVA test, and Tukey’s multiple comparisons test were performed to compare data between all groups. Correlations were determined using Pearson’s ρ correlation for data exhibiting approximate symmetry, and spearman correlation for data exhibiting skewness. All tests were two-sided and significance was determined at *p* < 0.05. GSE52237 selected was transformed for the analysis in Z-score intensity signal, in order to allow the comparison of data independently of the original hybridization intensities. Linear regression analysis was also performed to test the association between puff numbers/nicotine content and biomarkers of inflammation. Multivariate analysis was used to test the independent association between the variables of interest by adjusting for nicotine levels. Multiple regression analysis was also performed to identify the relationship of membrane ACE-2 expression levels with puff number, nicotine, IL8 and IL6 concentrations.

## Results

### Nicotine Concentration in Exposed Cell Culture Medium

The generation and delivery of cigarette smoke to the ALI systems was assessed by the quantification of nicotine in the cell culture medium (DMEM) from each chamber after the exposure of cells to smoke. The nicotine concentration increased in a dose-dependent manner according to the number of puffs with 2 (nicotine = 1.5 μg/ml), 5 (nicotine = 5.7 μg/ml), 10 (nicotine = 12 μg/ml), 15 (nicotine = 19.8 μg/ml) and 20 (nicotine = 31.4 μg/ml) puffs. Instead, the air control nicotine concentration was 0.2 μg/ml (Supplementary Figure S2).

### Membrane ACE-2 Expression on H292 Cells

**FIGURE 1:**
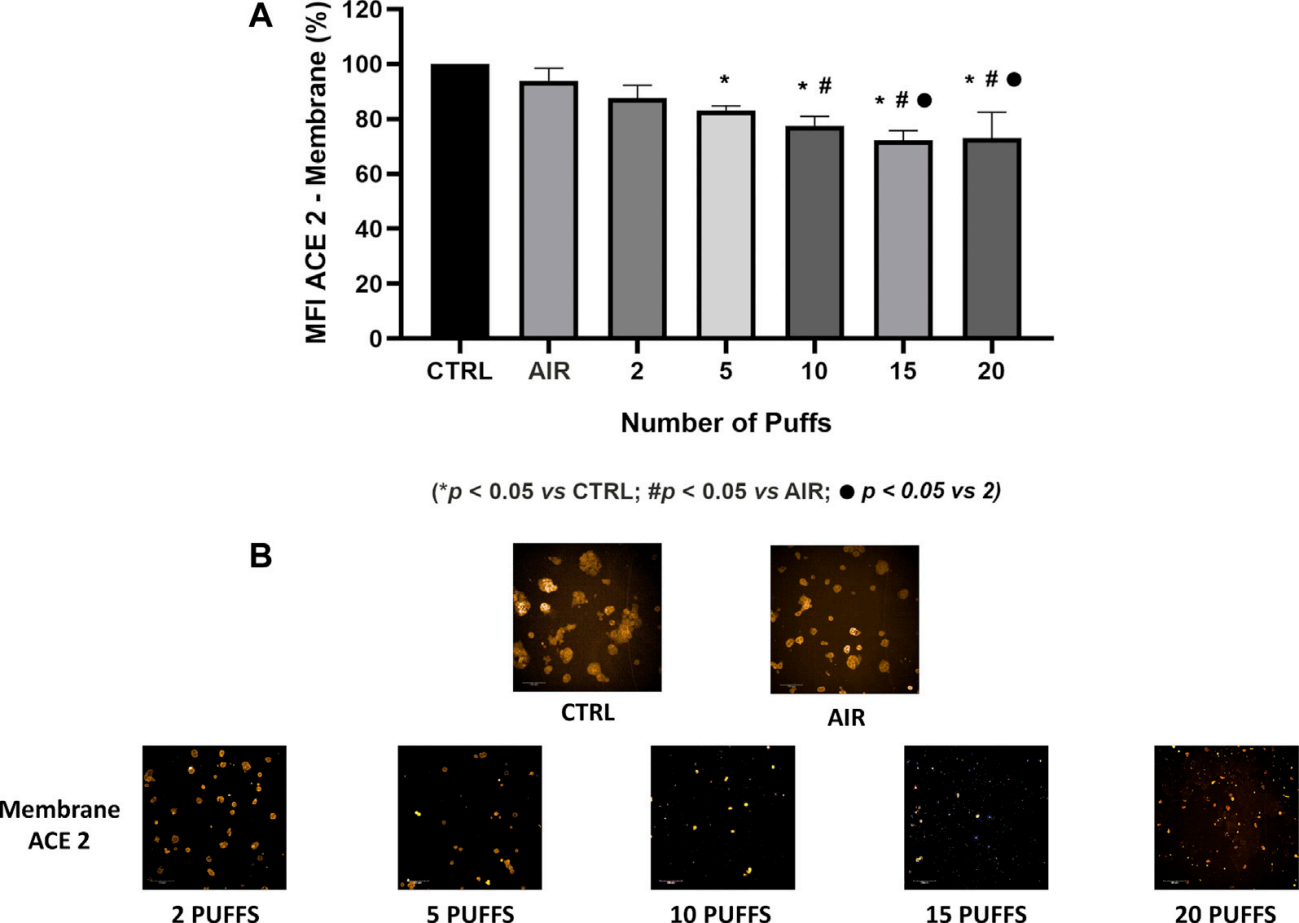
Quantification of membrane ACE-2 expression normalized to live cells, following membrane segmentation by HCS using the Harmony software **(A)**. Representative images of immunocytochemistry of membrane ACE-2 expression following exposure to increasing number of puffs of cigarette smoke **(B)**. Images were used for segmentation and protein expression quantification on cell membranes.

**FIGURE 2:**
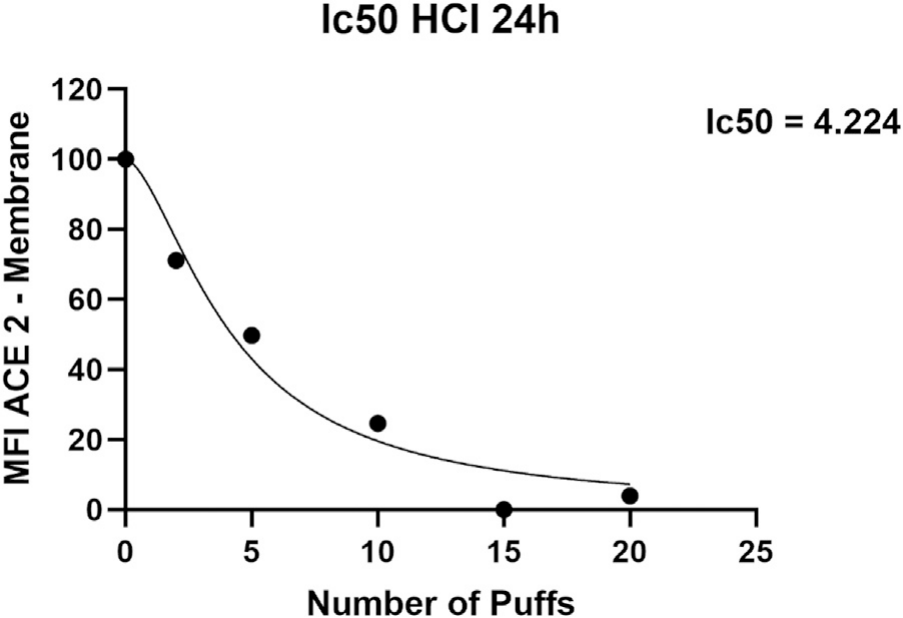
IC50 **(**half maximal inhibitory concentration) curve for ACE-2 expression obtained with increasing puff number of 1R6F smoked under HCI (Health Canada Intense) regime on H292 cells at Air-Liquid Interface (ALI).

Exposure to 1R6F cigarette smoke showed a dose dependent decrease of ACE-2 expression on H292 membranes after 24 h from the smoke exposure compared to air exposure and control (Figure 1). Particularly, we observed significant differences of membrane ACE-2 expression percentage in H292 cells exposed to 5, 10, 15, and 20 cigarette puffs compared to incubator control normalized to live cells (*p* values < 0.05). Moreover, the expression of membrane ACE-2 is reduced in cells exposed to 10, 15 and 20 cigarette puffs compared to AIR exposed control (*p* values < 0.05). The IC50 (half maximal inhibitory concentration) for the expression of membrane ACE-2 can be reached at about four puffs of cigarette 1R6F smoked under HCI regime (Figure 2).

### Concentration of IL6 and IL8 Inflammatory Cytokines

**FIGURE 3:**
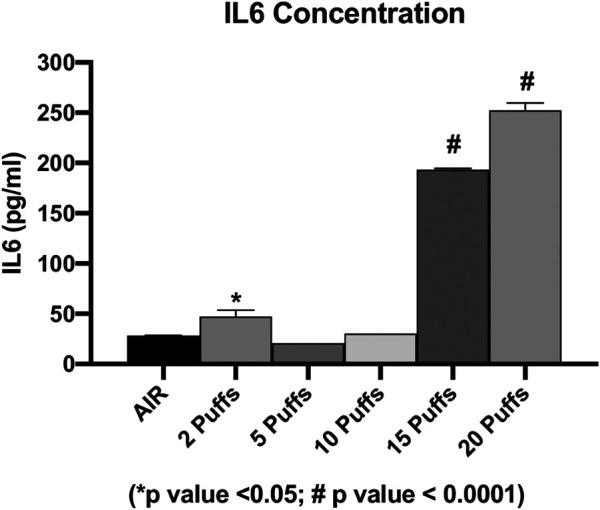
IL6 concentration as a function of puff number of cigarettes 1R6F smoked under HCI (Health Canada Intense) regime. *p* values were calculated between each puff number vs. AIR control.

**FIGURE 4:**
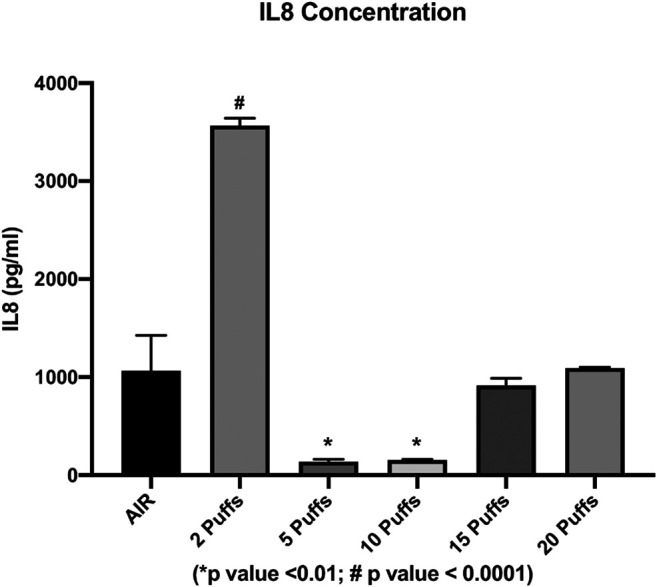
IL8 concentration as a function of puff number of cigarettes 1R6F smoked under HCI (Health Canada Intense) regime. *p* values were calculated between each puff number vs. AIR control.

The concentration of secreted IL6 and IL8 were evaluated in order to assess the cigarette smoke induction of inflammation. We observed a dose-dependent increase in the IL6 levels from AIR exposure to the maximum dose of exposure, except for the IL6 level released from cells exposed to two cigarette puffs (Figure 3). The measured values were 28.35 ± 0.2 pg/ml for AIR, 47.26 ± 6.3 pg/ml for two puffs, 20.89 ± 0.1 pg/ml for five puffs, 30.38 ± 0.1 pg/ml for 10 puffs, 193.32 ± 1.4 pg/ml for 15 puffs, 252.45 ± 7.1 pg/ml for 20 puffs. We observed a significant difference for 15 and 20 puffs compared to AIR control (*p* < 0.0001). Moreover, exposure with two puffs of cigarette smoke is significantly different from AIR control (*p* < 0.05). Instead, we observed a different profile of IL8 concentration compared to IL6 release (Figure 4). The higher mean ± SD value of IL8 was shown after two puff of cigarette smoke, 3,566.77 ± 74.63 pg/ml. This increase is significant compared to AIR control, 1,067.33 ± 358.3 pg/ml, with a *p* value < 0.0001. Whereas, the mean ± SD of IL8 concentrations after 5 (138.59 ± 23.11 pg/ml) and 10 puff (155.42 ± 6.92 pg/ml) of cigarette smoke were much lower compared to AIR control. Moreover, the mean ± SD of IL8 concentrations after 15 (914.68 ± 73.29 pg/ml) and 20 puff (1,093.58 ± 7.8 pg/ml) did not differ from AIR control.

### Correlation Analyses With Membrane ACE-2 Protein Expression

**FIGURE 5:**
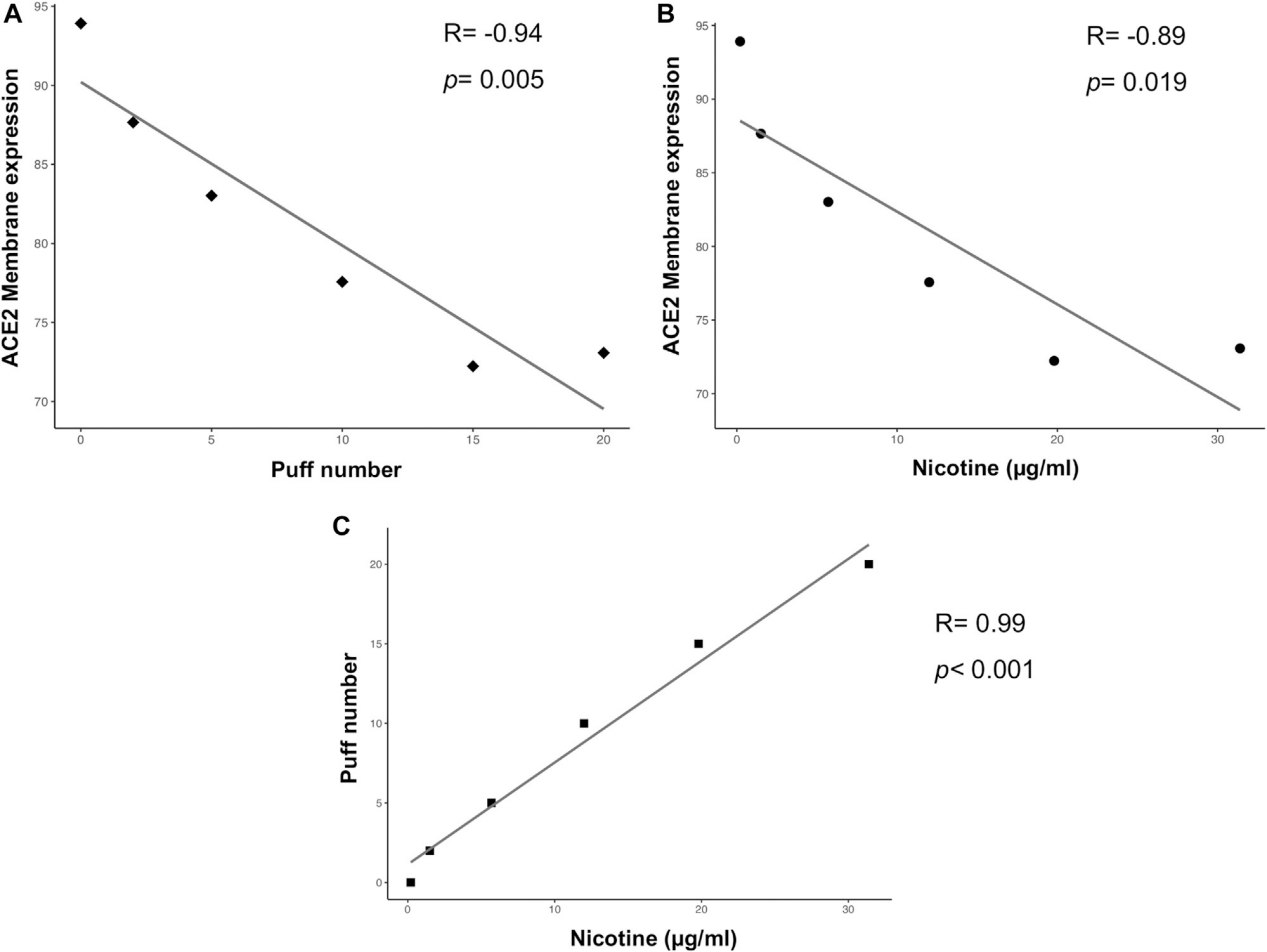
Scatter plots of correlation analyses of membrane ACE-2 protein expression on H292 cells *vs* puff number **(A)** and nicotine dosage **(B)**, and puff number *vs* nicotine dosage **(C)**. Correlations were determined using Pearson’s ρ correlation.

**FIGURE 6:**
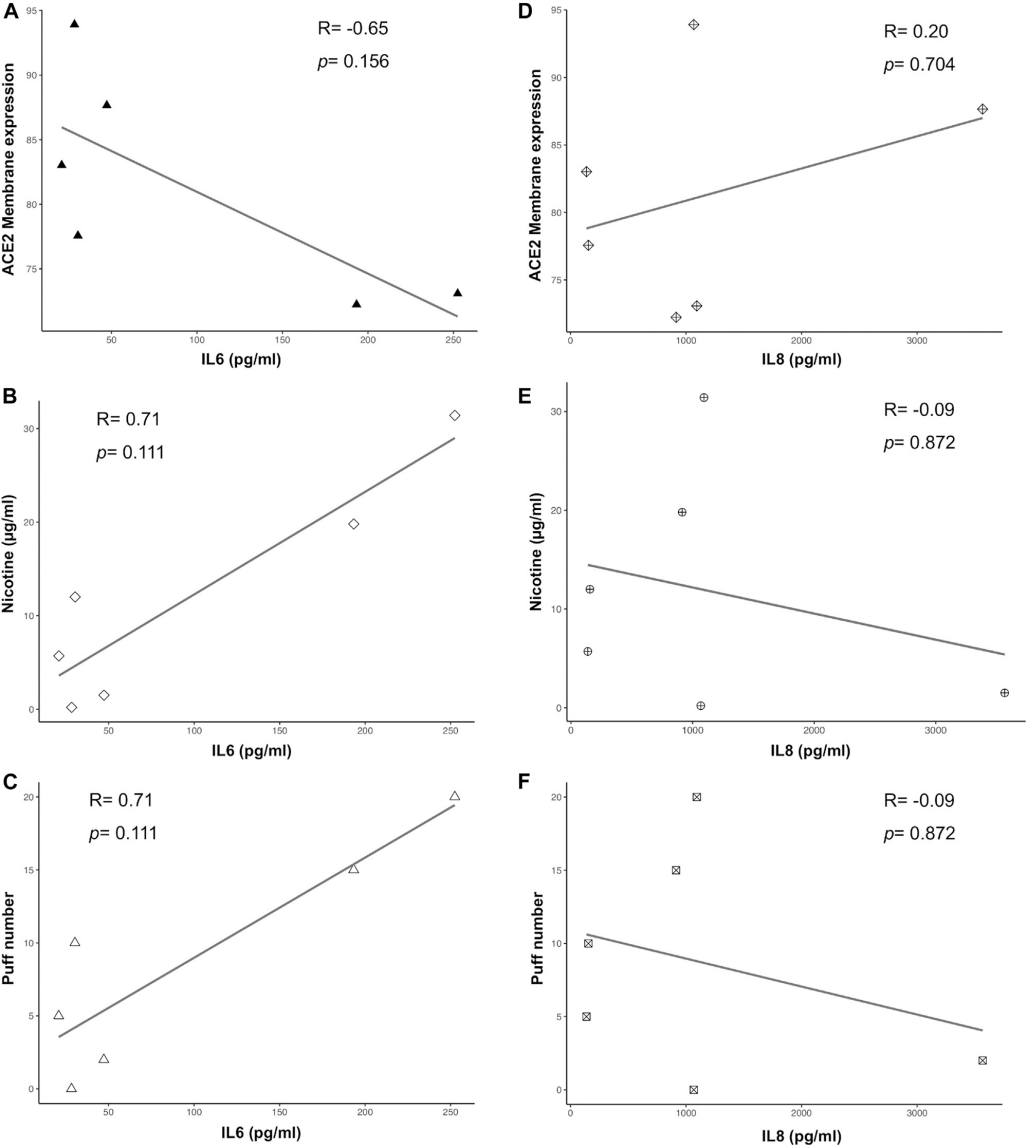
Scatter plots of correlation analyses of both IL6 **(A-C)** and IL8 **(D-F)** vs ACE2 membrane expression, puff number and nicotine dosage. Correlations were determined using Spearman correlation.

Multiple correlation analyses were performed to evaluate the relationship between ACE-2 expression and different parameters, including puff number, nicotine, IL6 and IL8 concentrations. Membrane ACE-2 expression correlated negatively with puff number (R = −0.94; *p* = 0.005) (Figure 5A) and nicotine concentration (R = −0.89; *p* = 0.019) (Figure 5B). Moreover, we observed a strong correlation between puff numbers and nicotine concentrations (R = 0.99; *p* < 0.001). Instead, both IL6 and IL8 concentrations did not correlate with any others parameters (Figure 6). Multiple linear regression analyses were also performed in order to verify the interaction of puff number, nicotine dosage, IL6 and IL8 concentrations with the membrane ACE-2 expression. Results indicates that changes in puff number and nicotine concentration are significantly associated with changes in membrane ACE-2 expression. Whereas, changes in IL6 and IL8 concentrations did not significantly affect membrane ACE-2 expression (Table 1).

**TABLE 1:** Multiple Linear regression analysis ACE2 expression vs puff number, nicotine, IL6, and IL8.

Regression summary					
*Count*	*Missing observation*	*R squared*	*Adjusted R squared*	*Residual Standard Error*	*Overall p value*
6	0	0.9916	0.9581	1.752	0.137
Regression coefficients					
	*Coefficient*	*Standard Error*	*t value*	*p value*	
*Intercept*	92.873	1.855	50.074	0.0127	
*Puff number*	−3.547	0.769	−4.611	0.1360	
*Nicotine*	1.608	0.591	2.722	0.2242	
*IL6*	0.0037	0.027	0.138	0.9129	
*IL8*	-0.0001	0.0009	−0.165	0.8959	
ANOVA					
	*DF*	*Sum of squares*	*Mean square*	*F value*	*p value*
*Puff number*	1	324.30	324.30	105.699	0.0617
*Nicotine*	1	38.87	38.87	12.667	0.1744
*IL6*	1	0.01	0.01	0.0020	0.9714
*IL8*	1	0.08	0.08	0.0272	0.8959
*Residuals*	1	3.07	3.07		

### Bioinformatic Analysis

**FIGURE 7:**
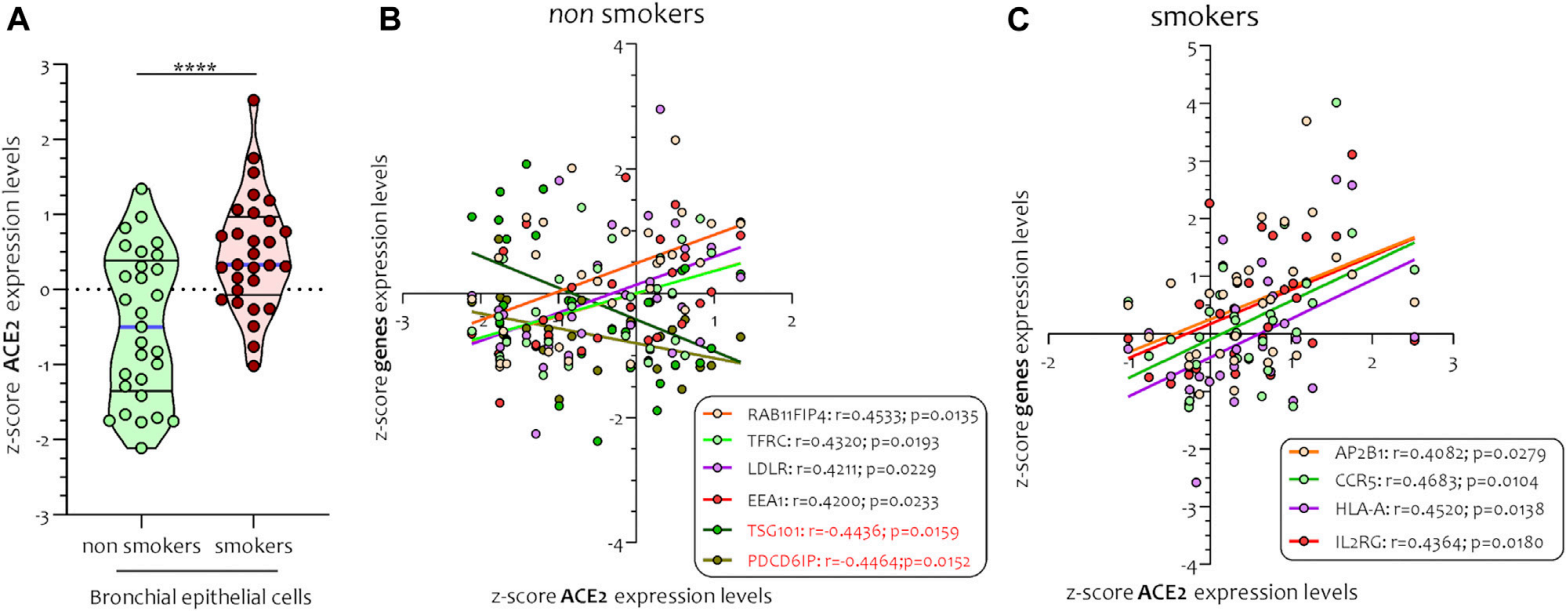
Expression levels of ACE2 **(A)** and Pearson’s correlation analysis between *ACE-2* and *nAChR (CHRNA7)* z-score *mRNA* expression levels in smokers **(B)** and non-smokers **(C)** small airways epithelial (SAE) cells. Data are expressed as dot plot.

**FIGURE 8:**
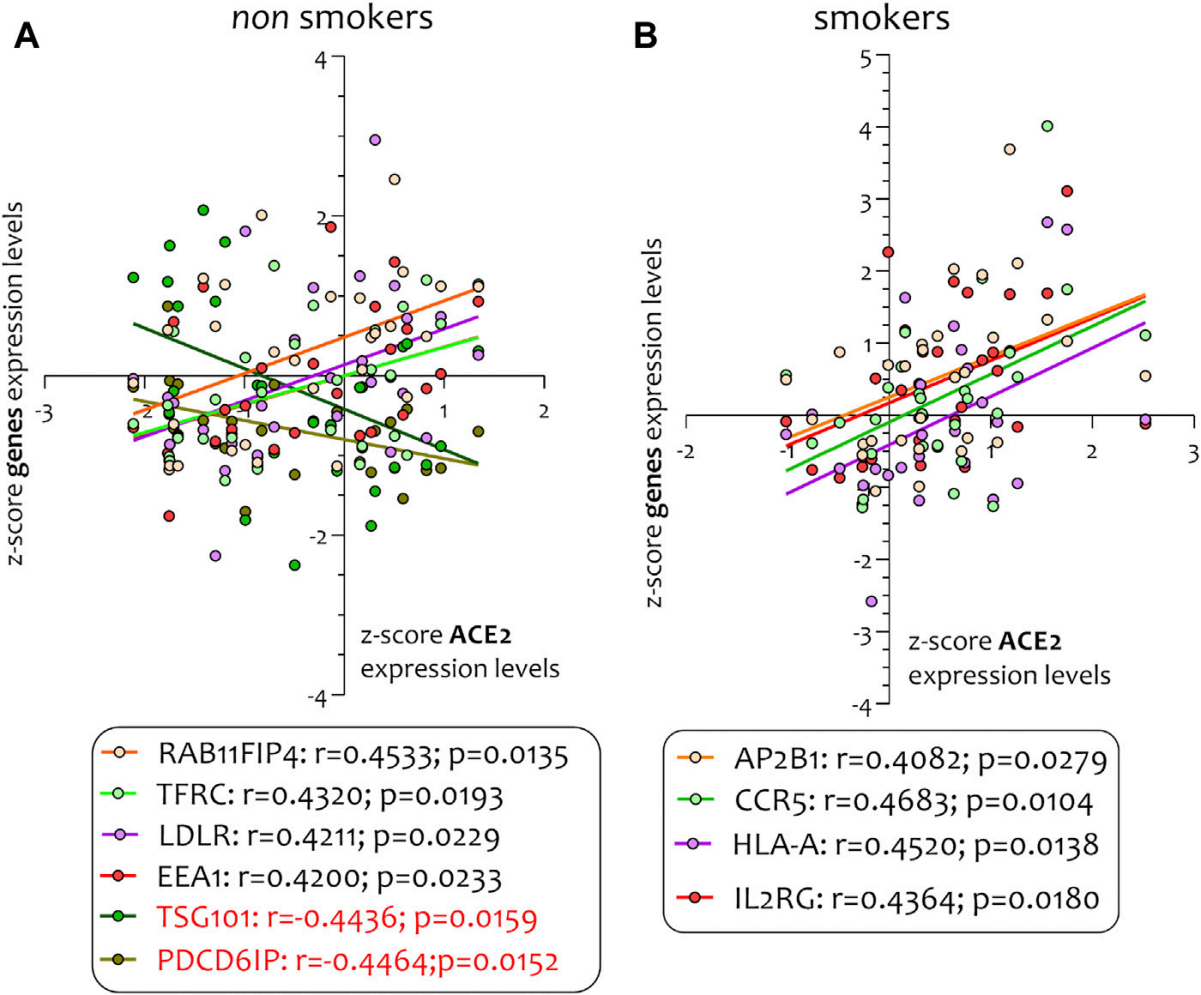
Pearson’s correlation analysis between *ACE-2* and its genes pathways in non-smokers (PDCD6IP, TSG101, RAB11FIP4, LDLR, TFRC, and EEA1) **(A)** and smokers (AP2B1, CCR5, HLA-A, and IL2RG) **(B)**.

**FIGURE 9:**
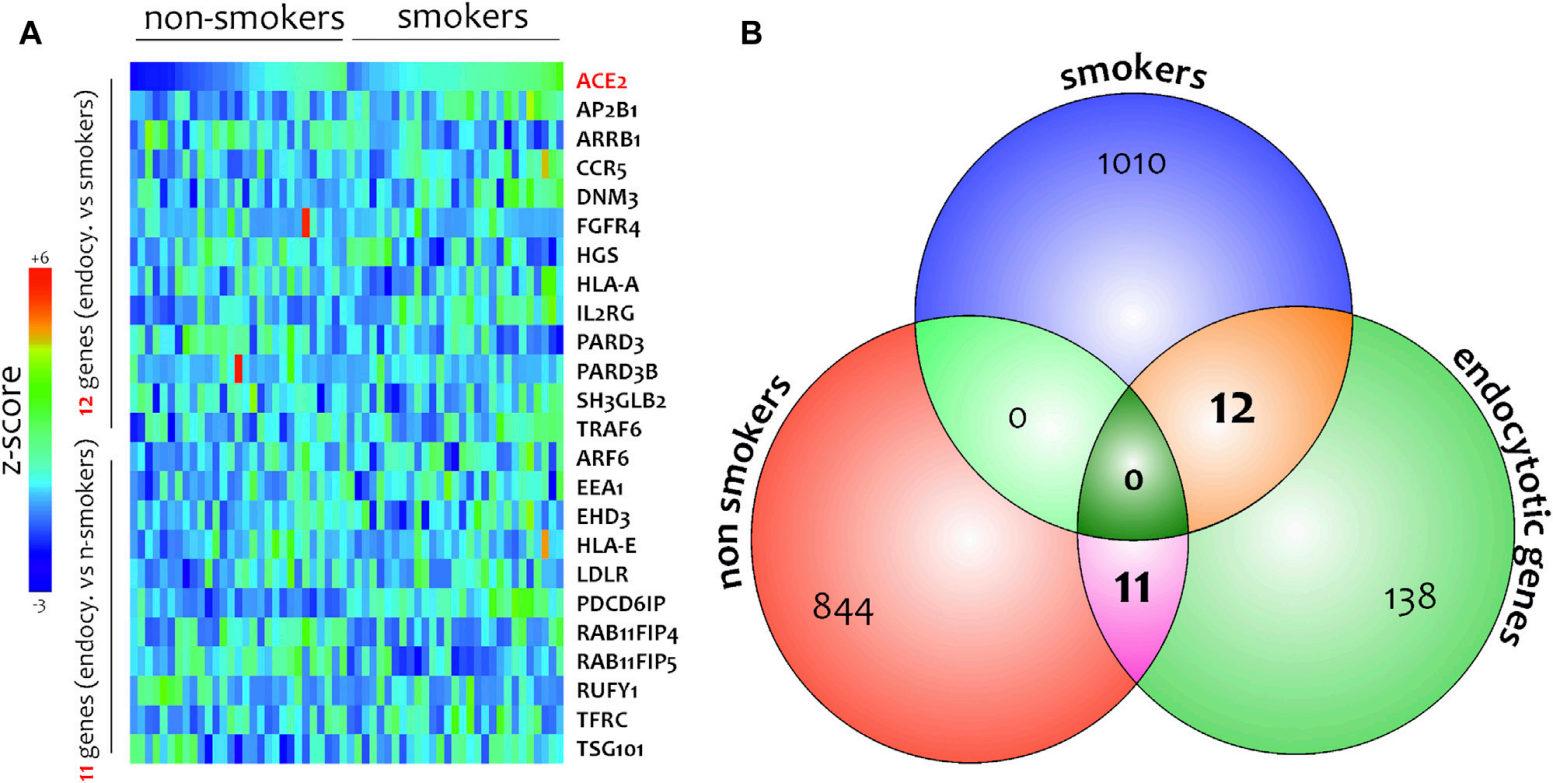
Heatmaps of genes highly related to ACE-2 in SAE cells isolated from non-smokers and smokers **(A)**. Venn diagram performed between gene highly correlated to *ACE-2* in non-smokers, smokers, and main genes belonging to *endocytic process* **(B)**.

**FIGURE 10:**
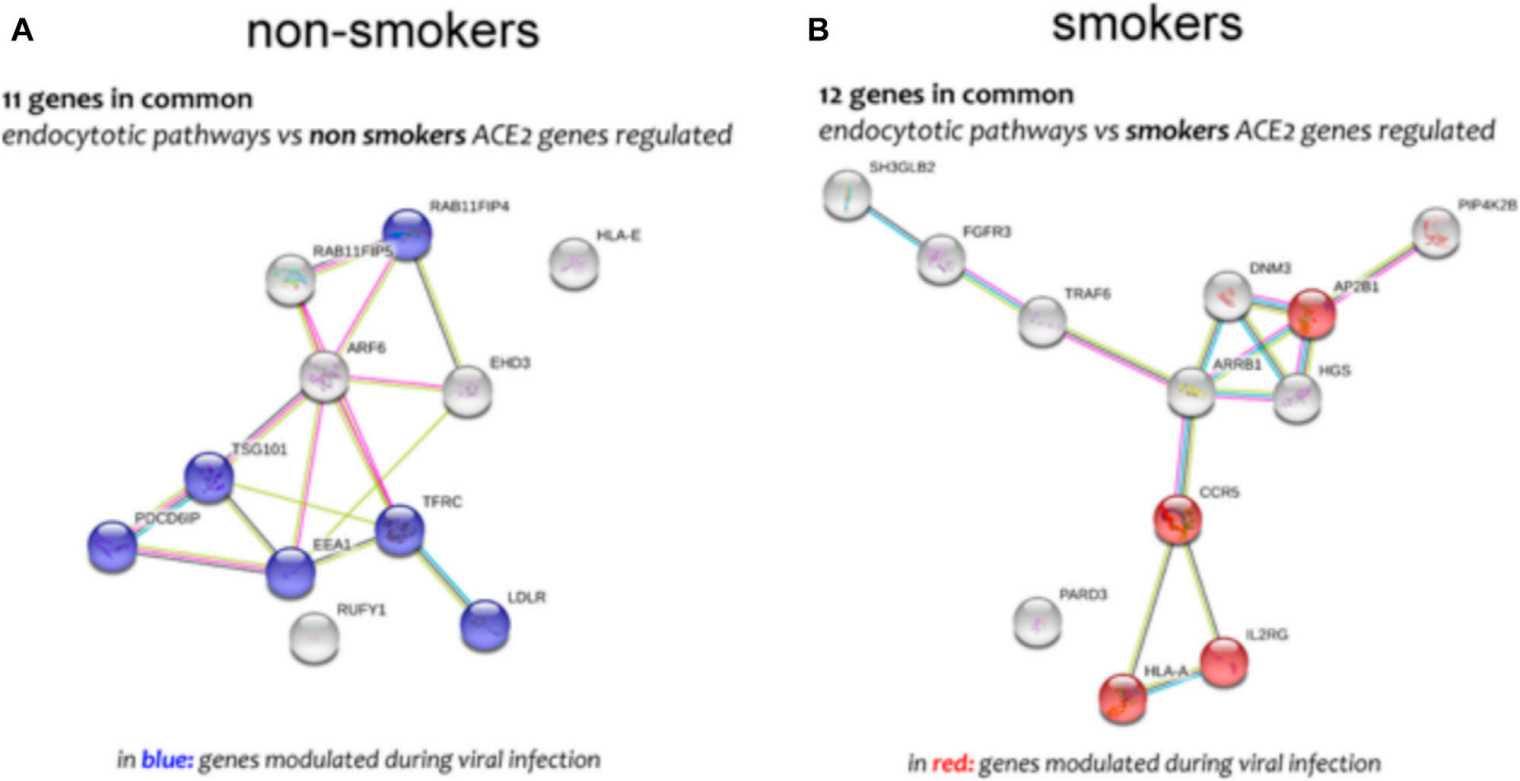
Genes network analysis of main genes highly correlated to *ACE-2* in non-smokers **(A)** and smokers **(B)** SAE cells, performed by online tools STRING.

Different airway cells, such as bronchial epithelial cells, type II alveolar epithelial cells, and interstitial fibroblasts lung, express nicotinic acetylcholine receptors (nAChR), specifically the α7subtype (Leung et al., 2020a). Consistently with these results our preliminary data based on GEO datasets of smokers and non-smokers subjects showed a significant increase of ACE-2 mRNA in small airway epithelium cells from smokers compared to non-smokers (Figure 7A) and an inverse correlation between nAChR (CHRNA7) and ACE-2 gene expression only in smokers (Figures 7B,C). Our preliminary results are based on microarray analysis of the GSE52237 dataset composed of small airway epithelium cells from smoker and non-smokers subjects. Our results further suggest that ACE-2 gene expression is differently correlated to other genes pathways in non-smokers (PDCD6IP, TSG101, RAB11FIP4, LDLR, TFRC, and EEA1), and smokers (AP2B1, CCR5, HLA-A, and IL2RG) (Figures 8A,B). By stratifying GSE52237 as a function of ACE-2 gene expression levels, we highlighted 1,012 genes related to ACE-2 in smokers and 855 in non-smokers. In order to verify a possible relationship between the genes co-expressed with ACE-2 and the genes involved during the endocytosis process, we performed a Venn diagram analysis. 161 genes involved in the endocytosis process (hsa04144) were highlighted using the online pathway tool KEGG (https://www.genome.jp/kegg/kegg2.html). The result showed that 11 genes were in common between the ACE-2 pathways in smokers and the genes regulated during endocytosis, while 12 genes with non-smokers (Figure 9). Among these genes, six in non-smokers and four genes in smokers were closely involved during the viral internalization process (Figure 10). Currently, there are very few studies on genes related to ACE2 expression. The genes expression analysis of ACE2 with ADAM17 in GSE52237, previously correlated with ACE2 (PMID: 15,983,030), did not show significant results (Supplementary Figure S3).

## Discussion

Coronaviruses use the homotrimeric spike protein (comprising S1 and S2 subunit in each spike monomer) exposed on the envelope to bind to the ACE-2 host cell receptors. Cryo-electron microscopy studies have shown that receptor binding induces the dissociation of the S1 with ACE2, prompting the S2 to transit from a metastable pre-fusion to a more-stable post-fusion conformation that is essential for membrane fusion (Leung et al., 2020b; Walls et al., 2020) and subsequent viral RNA entry into the cells. Therefore, binding to the ACE2 receptor is a critical initial step for SARS-CoV-2 entering into target cells.

Given these premises, it appears that ACE-2 expression on lung cells is a relevant issue in determining SARS infectivity in lung cells. Furthermore, several studies showed inconsistent results regarding the evidence that cigarette smoke may significantly regulate the expression of ACE-2 and thus resulting in a reduced SARS-Cov-2 risk of infection in smokers.

In an effort to verify this hypothesis, here we studied the effect of cigarette smoke on membrane ACE-2 expression on human bronchial epithelial H292 cells by an air-liquid interface system in order to achieve the best *in vitro* conditions resembling those of an *in vivo* exposure.

Our results with 1R6F reference tobacco cigarette showed a significant decrease on ACE-2 membrane protein expression. Moreover, we observed a significant correlation of ACE-2 expression on H292 membranes following cell compartment segmentation and nicotine concentration in the media or smoke puff number. However, it is also known that ACE-2 downregulation may be dependent on inflammatory mediators increased production (Hanafy et al., 2011) and therefore it was conceivable that our results could be merely an epiphenomenon related to such mechanism. To this regard, cigarette smoke promote inflammation inducing the production of pro-inflammatory cytokines, such a TNF-α, IL-1, IL-6, IL-8 and granulocyte-macrophage colony-stimulating factor (GM-CSF) (Cardinale et al., 2012).

Therefore, in order to exclude such eventuality, we quantified the release of the pro-inflammatory cytokine IL-6 and IL-8 from H292 cells exposed to smoke and air, correlating it to the membrane expression of ACE-2. We did not find a significant correlation between these values, confirming that the reduction of ACE-2 on lung cells may be a direct effect of nicotine or, otherwise, of some other component not yet identified in cigarette smoke. Our results are consistent with the rationale of a recent clinical trial (NCT04429815) aiming at demonstrating that subjects taking nicotine substitutes as part of a smoking cessation program are less infected with COVID-19 than non-smokers, that active smokers are less infected with COVID-19 than non-smokers and also at comparing the percentage of positive serological tests in subjects taking nicotine substitutes to the percentage of positive serological tests in active smokers.

We further investigated the possible molecular mechanisms related to reduced SARS-Cov-2 risk of infection and we performed a microarray analysis of the GSE52237 dataset composed of small airway epithelium cells of smokers and non-smokers. These set of experiments showed that in smokers and non-smokers who had ACE-2 high expression levels, there was a different transcriptional response due to molecular mechanisms inherent in processes of antiviral response and endocytosis. ACE-2 mRNA levels were correlated both in smokers and non-smokers with the gene expression specific to the two groups examined, all belonging to an antiviral response. These results suggest that cigarette smoking may act on the bronchial epithelium selectively, not by blocking a response on viral infection but simply by activating different genes that trigger different transcriptomic collateral mechanisms. This type of global transcriptomic view could provide us with an explanation of the possible collateral genomic events involved during viral infections such as SARS-COV2.

Consistently with previous reports we showed that ACE-2 gene expression was reduced in non-smokers compared to smokers. This apparent discrepancy with our protein expression profile may be dependent on the possibility that such upregulation is the result of a compensatory mechanism for the reduced expression of ACE-2 protein on the cellular membrane. Furthermore, the microarray dataset analysis does not take into due account the smoking habits of patients and their comorbidities, unfortunately not provided by the original authors of the dataset analyzed. Similarly, the same bias is applicable for all the other studies reporting the expression in bronchial biopsies which are not taking into due account eventual comorbidities of the enrolled patients. It should be noted that the observed decrease in ACE-2 expression in our study seems to be an acute effect of exposure of bronchial epithelial cells to smoke, whereas the data obtained from the analysis of long-term smoker biopsy samples show an increase of this receptor on the pulmonary epithelium. It could be hypothesized that in smokers the chronic exposure to cigarette smoke triggers a compensatory mechanism increasing the ACE-2 expression in order to balance the inflammatory effects induced by tobacco smoke in the long term.

Finally, one possible limitation of our study and of the others on this topic is related to the possible interaction of ACE-2 with the nicotine receptor nAChR. To this regard, nicotine, through α7-nAChRs present in neuronal and non-neuronal cells, has shown to upregulates ACE-2 (Wrapp et al., 2020) (Russo et al., 2020) Leung et al. (2020b) provided further evidence in support of this hypothesis and propose the use of α7-nAChR antagonists (e.g., methyllycaconitine, α-conotoxin) for the control of ACE-2 expression and to prevent SARS-CoV-2 entry. Consistently, Changeux et al. recently suggested a strong role for nAChR in the SARS-CoV-2 correlated disorders (Changeux et al., 2020). By virtue of previous observations on cryo-EM structure of the trimeric SARS-CoV-2 spike (S) protein, they hypothesized that SARS-CoV-2 virus itself is a nAChR blocker. Nicotine could then sterically or allosterically compete with the SARS-CoV-2 binding to the nAChR suggesting a protective role of this molecule against SARS-CoV-2 infection and the subsequent deficits it causes in the CNS. We did not perform experiments on nAChR since different nicotine receptors are present on bronchial epithelial cells (Strzelak et al., 2018) and the use of such antagonist would have been not conclusive.

Taken all together our data may offer a pharmaceutical role of nicotine as potential treatment option in COVID-19 patients. Furthermore, the outcomes of this study may provide new insights regarding the effect of nicotine on other coronaviruses intracellular entry and further understanding of the role of ACE-2 in the complex pathophysiology of cardiovascular and lung diseases in smokers.

## Data Availability

The datasets presented in this study can be found in online repositories. The names of the repository/repositories and accession number(s) can be found below: https://www.ncbi.nlm.nih.gov/geo/, GSE52237.
