# Supplementary material for: Role of Cigarette Smoke on Angiotensin-Converting Enzyme-2 Protein Membrane Expression in Bronchial Epithelial Cells Using an Air-Liquid Interface Model
Source: Front Pharmacol. 2021 Mar 30;12:652102. doi: 10.3389/fphar.2021.652102 (PMC8042260; doi:10.3389/fphar.2021.652102)
Supplement: Supplementary file 1 [file datasheet1.docx]

Supplementary Material

# Supplementary Data

Supplementary Material should be uploaded separately on submission. Please include any supplementary data, figures and/or tables. All supplementary files are deposited to FigShare for permanent storage and receive a DOI.

Supplementary material is not typeset so please ensure that all information is clearly presented, the appropriate caption is included in the file and not in the manuscript, and that the style conforms to the rest of the article. To avoid discrepancies between the published article and the supplementary material, please do not add the title, author list, affiliations or correspondence in the supplementary files.

# Supplementary Tables

**Supplementary Table 1.** MRM transitions monitored (m/z) with cone and collision voltages.

| **Analyte** | **MRM (m/z)** | **Cone (volts)** | **Collision energy (eV)** |
| --- | --- | --- | --- |
| Nicotine | 163 → 117 | 40 | 25 |
|  | 163 → 132 | 40 | 15 |
| Nicotine-(methyl-d3) | 165.8 → 116.8 | 40 | 20 |
|  | 165.8 → 129.7 | 40 | 20 |
|  |  |  |  |

## Supplementary Figures


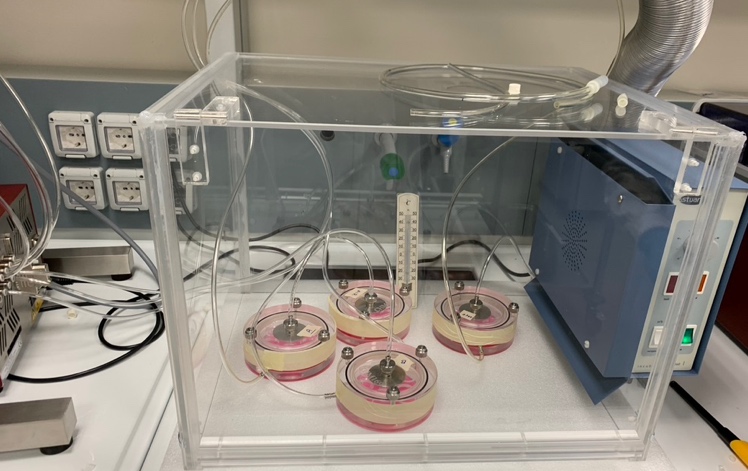

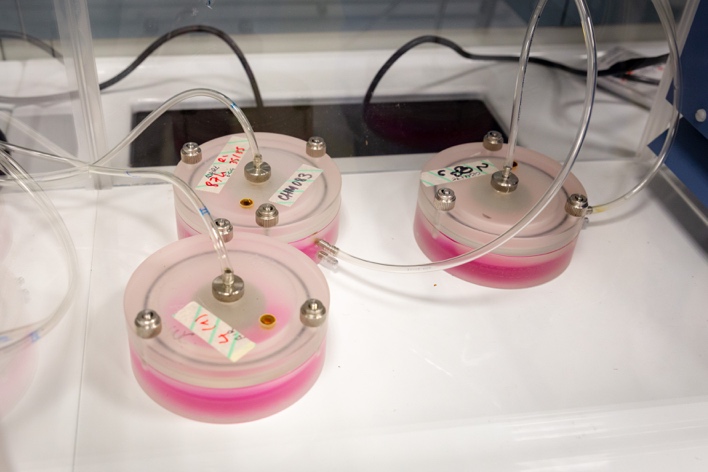


(A)

(B)

**Supplementary Figure 1. (A)** Exposure perspex chambers placed in a temperature controlled (37°C) total visibility incubator. **(B)** Exposure perspex chambers with the polyurethane transparent pneumatic tube overhead which carries the cigarette smoke from the smoking machine on the top of the cells and the lateral tube which slowly removes the smoke from the lower part of the cells, allowing a consistent exposure, but avoiding the accumulation of smoke inside the system.


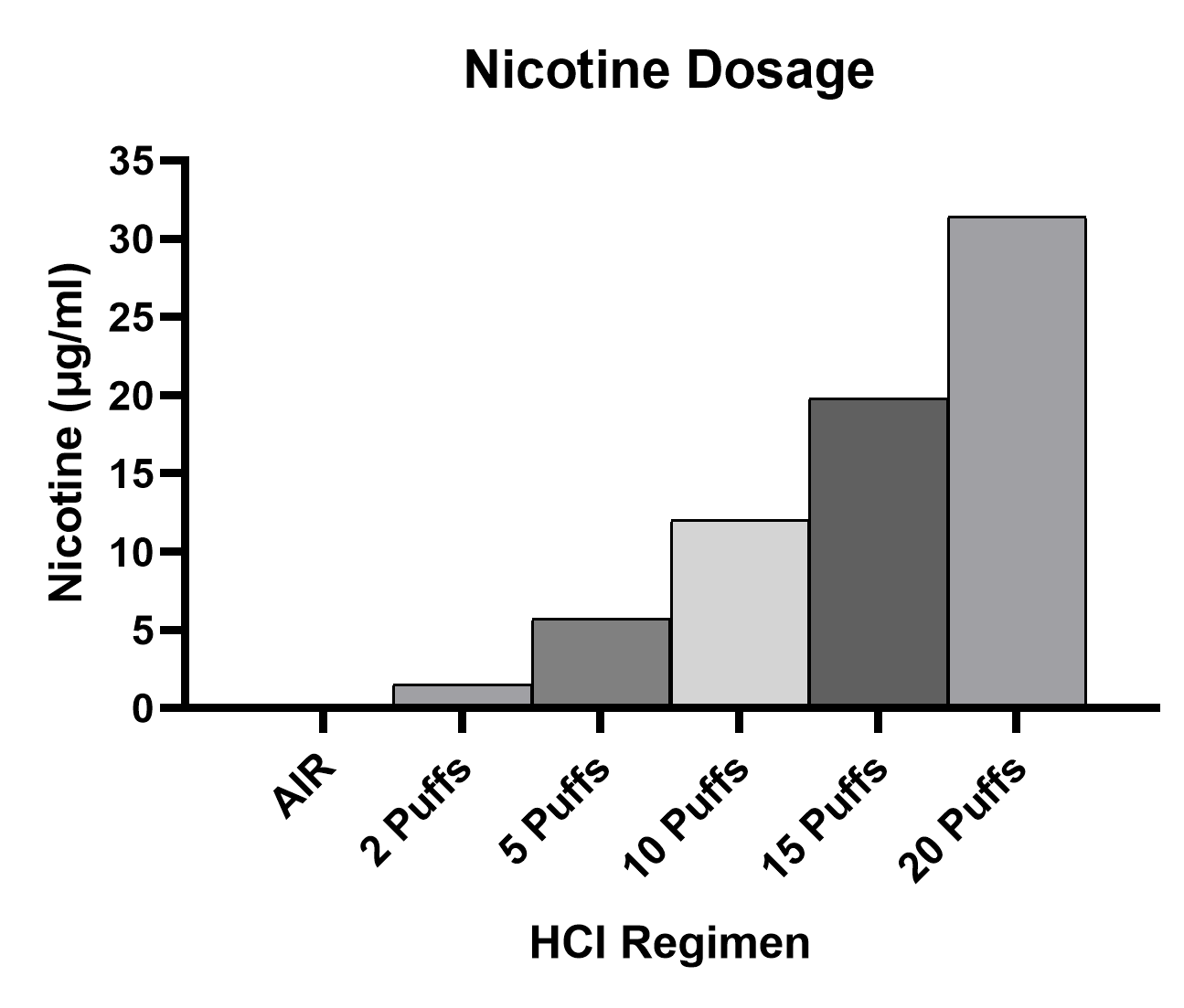


**Supplementary Figure 2.** Nicotine dosage in cell culture media of the above presented experiments with increasing puff numbers of cigarette 1R6F smoked under Health Canada Intense (HCI) regime.


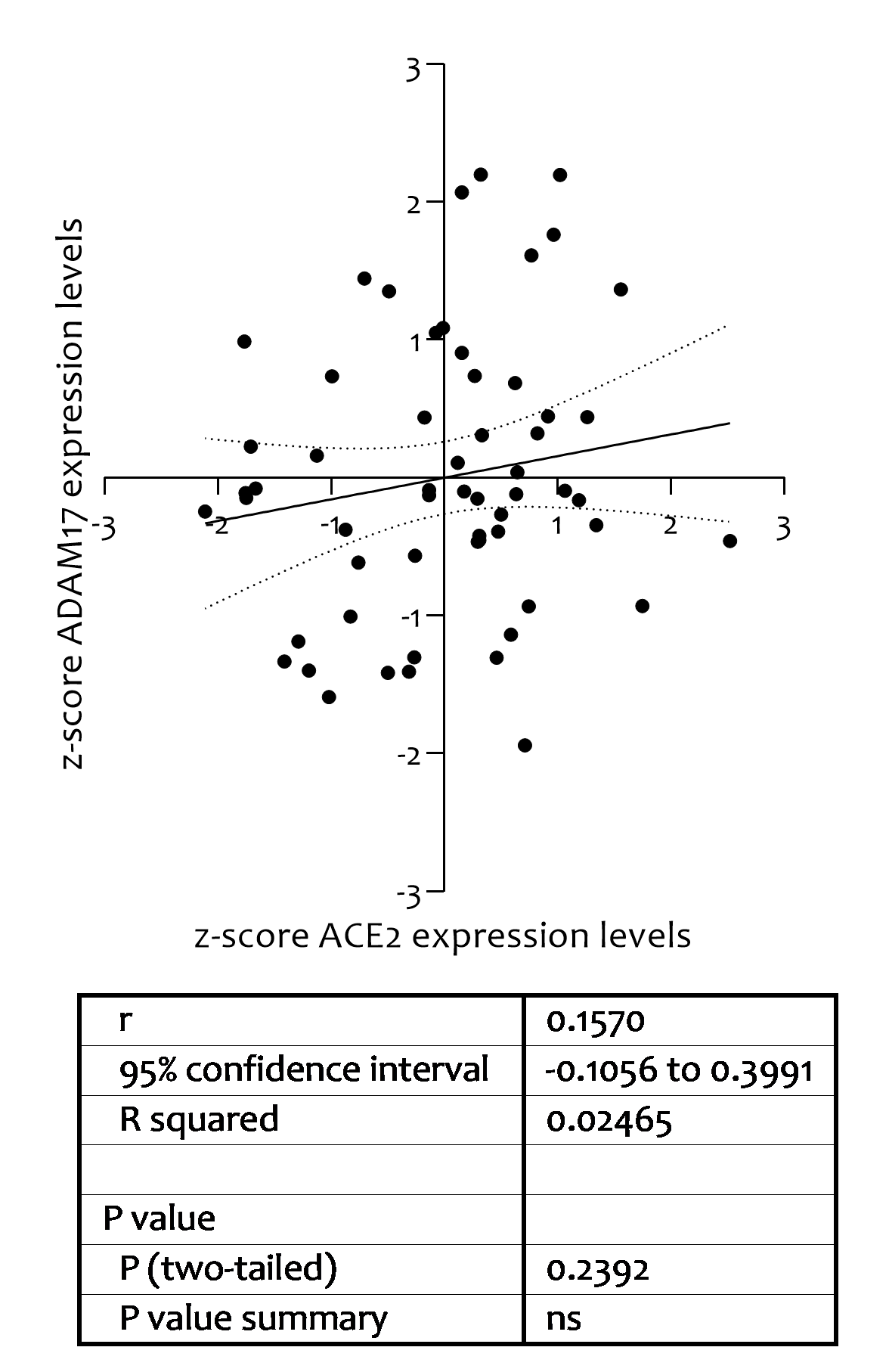


**Supplementary Figure 3.** Correlation analysis of ACE2 and ADAM17 genes. There are not significative correlations in our cells model (P = 0.2392).
